# The South American Dung Beetle Genus *Ennearabdus* Lansberge (Coleoptera: Scarabaeidae: Scarabaeinae: Eucraniini)

**DOI:** 10.1673/031.010.9301

**Published:** 2010-07-05

**Authors:** Federico C. Ocampo

**Affiliations:** Instituto Argentino de Investigaciones de las Zonas Áridas, CCT-CONICET, Mendoza. CC 507. 5500. Mendoza, Argentina

**Keywords:** Argentina, conservation, lectotype, Monte, systematics

## Abstract

The South American endemic dung beetle genus *Ennearabdus* Lansberge is revised. Description, diagnosis and illustrations are presented for the only known species of the genus, *E. lobocephalus* (Harold). A lectotype is designated for *Onthophagus lobocephalus* Harold, the type species of *Ennearabdus*. The biology, biogeography, conservation status, and distribution based on the predictive distribution model of *E. lobocephalus* are also discussed.

## Introduction

The genus *Ennearabdus* Lansberge is a monotypic endemic of the Argentinean North Western region that is rarely collected (probably because the area is not frequently visited by entomologists) and is consequently rare in collections. This genus is a member of the tribe Eucraniini, a relatively small tribe of dung beetles currently with four genera that is endemic to Argentina. The systematic placement of the genus within dung beetle classifications has been enigmatic and has changed numerous times. The only known species, *E. lobocephalus* (Harold 1868), was originally placed in the genus *Onthophagus* Latreille (Onthophagini). Later, Lansberge (1874), described the genus *Ennearabdus* and indicated that the genus was related to the Phanaeini as a “transition form” between them and the “Coprides”(i.e, *Copris* Geoffrey, *Dichotomius* Hope). Since Lansberge (1874), the genus was placed in catalogs as a Phanaeini (Gillet 1911; Bruch 1911; Blackwelder 1944). Olsoufieff (1924) did not treat the genus in his revision of Phanaeini. Later, Pereira and Martinez (1956) considered that there was not enough justification to keep *Ennearabdus* in Phanaeini and described the tribe Ennearabdini for this monotypic genus, but they did not indicate its phylogenetic relationships. Zunino (1983, 1985) was the first author to indicate the relationship between *Ennearabdus* and the tribe Eucraniini, at that time composed of three genera, *Eucranium* Brullé, *Anomiopsoides* Blackwelder, and *Glyphoderus* Westwood. Philips et al. (2002) and Ocampo and Hawks (2006), in their phylogenetic analysis based on morphological and molecular data, respectively, proposed a close relationship of the four Eucraniini genera and its sister group, the Phanaeini. Zunino et al. (1993), Monteresino and Zunino (2003), and Ocampo and Hawks (2006) described various aspects of the biology and behavior of *E. lobocephalus*.

The purpose of this contribution is to provide a taxonomic revision of *Ennearabdus*, and discuss this species' biology, biogeography, and conservation status.

## Material and Methods

Body measurements, puncture density, puncture size, fovea density, fovea size, and density of setae are based on the following standards. Body length was measured from the apex of the pronotum (at the middle) to the apex of the elytra, head is excluded and measured separately because the variable position of the head and length of clypeal teeth render it impractical to include in the body length). Body width was measured across mid-pronotum. Puncture density was considered “dense” if punctures were nearly confluent to less than two puncture diameters apart, “moderately densely foveate” if punctures were two to six diameters apart, and “sparse” if punctures were separated by more than six diameters apart. Puncture size was defined as “small” if punctures were < 0.02 mm in diameter, “moderate” if 0.02–0.07 mm in diameter, and “large” if > 0.07 mm in diameter. Surface was defined as “sparsely foveate” if there was (on average) a space of more than one diameter between foveae, “moderately dense” if there were 0.5–1.0 diameters between foveae, and “densely foveate” if foveae were confluent or separated by less than 0.5 diameters. Setae were defined as “sparse” if there were few setae and surface is distinctively visible, “moderately dense” if the surface was visible but with many setae, and “dense” if the surface was not visible through the setae. Elytral carinae were counted from the elytral suture. Specimen labels were copied literally using a “/” between lines.

Lectoypes are here designated to provide the nomenclatural stability of the taxon studied, according to Article 72 of the International Code of Zoological Nomenclature.

Specimens for this research were collected, borrowed from and deposited in the following institutions and collections.

CMNC: Canadian Museum of Nature, Ottawa, Canada (RS Anderson, F. Génier).
IAZA: Instituto Argentino de Investigaciones de las Zonas Áridas, Mendoza, Argentina (S Roig-Juñent, FC Ocampo).
MNHN: Muséum National d'Histoire Naturelle, Paris, France (O Montreuil).
UNSM: University of Nebraska State Museum, Lincoln, NE, USA (BC Ratcliffe, ML Jameson-Russell).
USNM: United States National Museum, Washington D.C. USA (D Furth).

### Predictive models of species distribution

Species distribution models are used to predict species potential distribution by relating known species collection localities to a set of environmental variables that, presumably, reflect the ecological niche of the species (Guisan and Thuillier 2005). Known localities for *E. lobocephalus* were georeferenced and mapped to model its distribution using predictive methods based on bioclimatic variables. MaxEnt (Phillips et al. 2006) was used combined with 19 bioclimatic variables obtained from WorldClim dataset (Hijmans et al. 2005). The resolution of the environmental layers was approximately 4.6 × 4.6 km.

***Ennearabdus* van Lansberge 1874 (Figures 1–16)**
Type species: *Onthophagus lobocephalus* Harold 1868: 84, by monotypy.

***Ennearabdus lobocephalus* (Harold 1868)**
*Onthophagus lobocephalus* Harold 1868. (original combination)

### Type material:

**Lectotypes.** Lectotype at MNHN labeled: “Mendoza;” “lobocephalus / Harold;” “Ex. Musæo / E. Harold;” “Muséum Paris / ex coll / R. Oberthür / 1952;” “*Ennearabdus lobocephalus* / det: F. C. Ocampo / ID: FCO5062;” “*Onthophagus lobocephalus* / Harold / Lectotype / F. Ocampo det.” (red label, handwritten).

**Paratypes.** One paralectotype at MNHN with same label as lectotype except: “*Ennearabdus lobocephalus* / det: F. C. Ocampo / ID: FCO5063;” “*Onthophagus lobocephalus* / Paralectotype / F. Ocampo det.” (yellow label, handwritten). One paralectotype at IADIZA labeled: “Mendoza / lobocephalus / Har.;” Museum Paris / coll. H. W. Bates / 1952;” “Museum Paris / ex coll. / R. Oberthür / 1952;” “*Ennearabdus lobocephalus* / det: F. C. Ocampo / ID: FCO5064;” “*Onthophagus lobocephalus* / Paralectotype / F. Ocampo det.” (yellow label, handwritten). Four paralectotypes at MHNH and one at IADIZA labeled: “Ex. Musæu / E. Harold”; “Museum Paris / ex coll. / R. Oberthür / 1952;” “*Ennearabdus lobocephalus* / det: F. C. Ocampo / ID: FCO5065” (and sequential numbers: FCO5066–69). “*Onthophagus lobocephalus* / Paralectotype / F. Ocampo det.” (yellow label, handwritten). One paralectotype at MNHN labeled: “Ex. Musæu / E. Harold;” “Museum Paris / ex coll. / R. Oberthür / 1952;” “*Ennearabdus lobocephalus* / det: F. C. Ocampo / ID: FCO5070;” “*Onthophagus lobocephalus* / Paralectotype / F. Ocampo det.” (yellow label, handwritten).

**Diagnosis.** *Ennearabdus lobocephalus* can be recognized from other members of the tribe by the hind wings fully developed (obsolete in the other genera), the metasternum relatively wide between mesocoxae (narrow in the other genera); and meso- and metatarsi with tarsal claws present, although reduced (tarsal claws absent in the other genera). The genus *Ennearabdus* can be recognized from the Phanaeini genera, to which Eucraniini is the sister taxon, by the meso- and metatibiae slender, expanded at apex and the meso- and metatarsal claws developed. The genus *Ennearabdus* can be recognized from South American Dichotomiini genera by the meso-and metatibiae slender, the metasterno gibbose, and the protarsi not developed.

**Figure 1.**
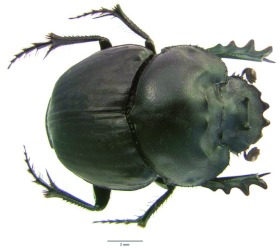
*Ennearabdus lobocephalus*, male. High quality figures are available online.

**Figures 2–8.**
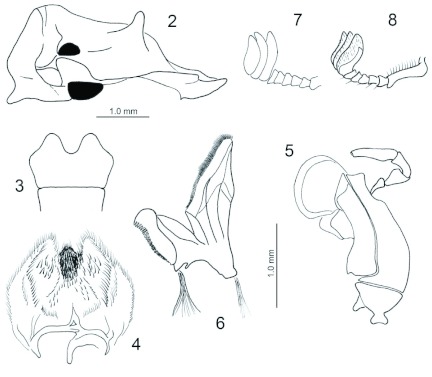
*Ennearabdus lobocephalus*. 2: head lateral view; 3: labium, ventral view, (setae and labial palps not illustrated); 4: labrum, ventral view; 5: left maxillae, ventral view (setae not illustrated); 6: left mandible, ventral view; 7: left antenna, dorsal view (setae not illustrated); 8: left antenna, ventral view. High quality figures are available online.

**Figures 9–12.**
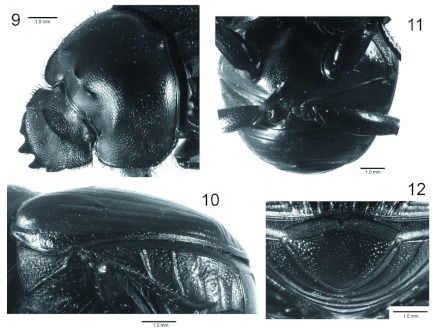
*Ennearabdus lobocephalus*. 9: female head and pronotum; 10: elytron and venter, lateral view; 11: metathorax and abdominal ventrites; 12: pygidium. High quality figures are available online.

**Redescription.** Male. Body length 7.56–10.80 mm, width 6.13–8.78 mm, head length 3.37–4.19 mm. (*n* = 78). *Color*: head, pronotum and elytra dull to shiny black, rarely with metallic green reflections; venter shiny black. *Head* (Figures 1, 2, 9): Frons convex, surface punctate at apex to rugopunctate at base. Paraocular area slightly convex, surface densely punctate, with small, reflexed tooth at apex. Postocular lobes of parietal depressed transversely (Figure 2). Cephalic carinae well developed, with 2 simple horns, horns variable in length (Figures 1, 9). Eyes small, completely divided, dorsal and ventral half not dorso-ventrally aligned. Canthal area distinct, slightly concave (Figure 2). Clypeus transverse; surface densely rugose (net-like), punctures large, clypeal anterior border smooth, with fringe of short setae, quadridentate, reflexed; medial teeth larger than lateral teeth, teeth separated by U-shaped incision (Figure 3); ventral surface densely punctate near margin, sparsely punctate on rest; ventral process well developed (narrow, not carina-like). Labium ventral surface densely setose, setae black, long; anterior margin U-shaped, lateral margins slightly angled; labial palp with 3 palpomeres, palpomere 1 dilated apically, palpomeres 1–2 densely setose, segment 3 glabrous; glossal surface smooth, without thick mat of setae; medial lobe of hypopharynx with transverse ridge of setae; lateral labial sclerites well developed, lateral arms of hypopharingeal Suspensorium as long as dorsal arm; oral arms not fused at middle, shorter than lateral arms. Labrum (Figure 4) ventral surface, with medium brush densely setose, setae short, thick; becoming sparse on disc; lateral files well developed; apical margin W-shaped, lateral margins setose, setae continuous with apical fringe, slender. Maxillae (Figure 5) articular process of cardo poorly expanded at apex, external surface setose, setae long; stipital sclerite II surface sparsely setose, setae short, slender; stipital sclerites I, IV densely setose, setae long; galea without channels at the base; articular sclerites well developed; maxillary palpi 4-segmented, segment 1, 2 subtriangular; 3, 4 subcylindrical; 4 as long as 2, 3 combined. Mandible (Figure 6) molar lobe with serrate area on ventral half, incisory lobe membranous surface setose, setae minute; incisor lobe prostheca with short setae on basal half, long setae at apical half. Antennae (Figures 7, 8) with 9 antennomeres, scape elbowed at base, antennomeres 2–6 conical, short; antennal club longer than wide, lamellae with apex acute, surface tomentose except medio-anterior portion of first lamella. *Pronotum* (Figures. 1, 9): Anterior portion rounded, membrane well-developed; antero-lateral and lateral portion broadly rounded, lateral portion bearing small irregular denticles; posterior angle rounded, slightly incised, posterior margin rounded, slightly protruded at middle. Surface rugo-punctate on sides and margin of disc to punctate on middle of disc, convex. Anterior half with 2 concave areas separated by convexity in middle, convexity with 2 poorly developed ridges near pronotal disc. Posterior pronotal fossae well-developed; lateral fossa developed. All pronotal margins beaded. *Elytra* (Figures 1, 10): convex, 0.66 times as long as wide, surface densely micropunctate (visible only at > 40 ×), sparsely punctate, punctures small; with 9 striae (excluding adjacent to epipleuron), striae 8–9 not reaching humeral angle. Pseudoepipleuron not developed. Epipleuron well-developed (Figure 10). *Hind wings:* Well developed. *Venter:* prosternum slightly carinate in middle, propleurum anteriorly and posteriorly punctate, sparsely punctate, setose; lateral margin densely setose, setae recumbent. Mesosternum short.

Metasternum broad, raised, gibbose; gibba conical, apex pointed (Figure 11). Metepisternum with base ∼2 times wider than apex, surface setose, setae long, moderately dense. Ventrites surface micropunctate at middle to punctate at sides. Pygidium (Figure 12) with base grooved medially; disc slightly convex, sparsely punctate, punctures moderate in size. *Legs* (Figure 1). Protibia with 4 lateral teeth, anterior protibial carinae well-developed, setose; protibial spur curved. Protarsi not developed. Meso-, metafemora longer then meso-, metatibiae, respectively. Meso-, metatibiae slender, apex expanded; surface setose; setae long, slender. Mesotibial spurs developed, inner spur ∼2 × longer than outer spur. Meso-, metatarsi well developed, becoming shorter from 1–4, 5 longer than 4. Meso-, metatarsal claws present; claws small, curved. Metatibial externo-dorsal margin denticulate, each denticle bearing seta.

**Figures 13–14.**
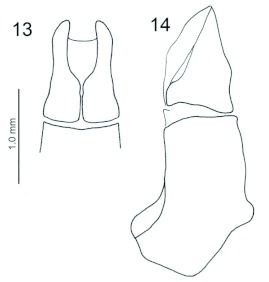
*Ennearabdus lobocephalus*, male. 13: parameres; 14: edeagus lateral view. High quality figures are available online.

Metatibial spur longer the first tarsomere. Male genitalia (Figures 13, 14): phallobase longer then parameres, symmetrical.

Female (Figure 9). Females are similar to males except on their cephalic armature: cephalic carinae less developed and lacking horns; and the pronotum anterior half with poorly developed concave areas separated by small convexity in middle, convexity with 2 poorly developed ridges near pronotal disc.

Minor males have well developed concave areas on anterior half of pronotum and poorly developed cephalic horns.

**Figure 15.**
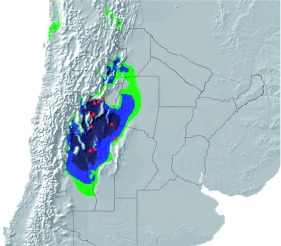
Map of distribution of *Ennearabdus lobocephalus* (red dots), and predictive distribution probabilities (green = 0.4–06, blue = 0.6–0.8, and dark blue = 0.8–1) of occurrence of the species based on 19 environmental variables. Values indicate probability of presence of the species in the area: 1 = present in the area, 0 = should not occur in the area. High quality figures are available online.

**Distribution** (Figure 15). Number of individuals is indicated in parenthesis. **ARGENTINA:** no data (7). **Catamarca:** Andalgalá (1); Andalgalá 36 km W (1); Esquiú (1); La Ciénaga, Belén (3); Rio Potrero (65 km NE Andalgalá) (3). **Córdoba:** Guanaco Muerto (2); **La Rioja:** Aimogasta (10 km E. Ruta Prov. 60) (5); Anillaco (2 km N, RN 75) (29); Chepes (1); Capital (La Rioja) (2); Mascasín (17); **Mendoza:** no more data (3). **San Juan:** Marayes (2).

Temporal distribution. January (17), February (31), March (2), November (1), December (2).

**Biology.** *Ennearabdus lobocephalus* shows typical tunneling behavior (Zunino 1983; Ocampo 2007) and is attracted to fresh and semi-fresh dung of large mammals, such as that from cow, human, and canids, or dry goat pellets (Martínez 1959). Specimens of *E. lobocephalus* were also observed flying and digging their burrows close to “cuis” nests (*Galea musteloides* Meyen) and lifting and carrying dry, dung pellets of this species with their fore legs to their previously dug burrows (Ocampo, personal observation). The behavior of digging the burrow before the storage of food is also characteristic of the other three genera of the tribe Eucraniini. Brood balls are pear-shaped and with a small cavity where probably the egg is laid (TK Philips, personal communication). Some aspects of the nesting behavior of *E. lobocephalus* were described by Monteresino and Zunino (2003). *Ennearabdus lobocephalus* were collected with dung traps baited with cow and horse dung. Based on personal observations, the species has diurnal activity, and they were not collected at lights (UV and MV).

**Phylogenetic relationships.** The genus *Ennearabdus* is related to *Eucranium* and a clade composed by *Anomiopsoides* and *Glyphoderus* (Philips et al. 2002; Ocampo and Hawks 2006; Monahan et al. 2007). Although phylogenetically related, *Ennearabdus* does not resemble a eucraniine morphologically; its gestalt appearance is more similar to a phanaeine. *Ennearabdus* presents several plesiomorphic morphological characters states within the Eucraniini: i.e, hind wings fully developed and functional; tarsal claws present; metasternum wide and raised (Ocampo and Hawks 2006).

**Figure 16.**
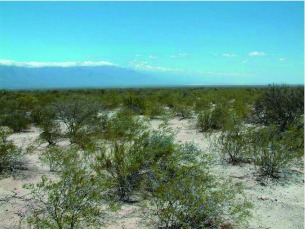
Collecting site showing the habitat of *Ennearabdus lobocephalus* in La Rioja province, Argentina. High quality figures are available online.

Biogeography and distribution. *Ennearabdus lobocephalus* is restricted to the Monte biogeographic province (Figures 15, 16). The Monte biogeographic province is a warm desert between Salta (24° 35′ S) and Chubut (43° 26′ S) provinces in Argentina (Morello 1958), limited by the Puna (north), Patagonia (south), Pampaena and Chacoan (east) biogeographic provinces, and the Andes (west). Patagonia and Puna have a related fauna and flora, whereas the Monte fauna and flora are more closely related to those of the Pampa and Chacoan provinces (Ringuelet 1961). Some Patagonian elements are also present in the Central and southern part of the Monte (Roig et al. 1980; Roig-Juñent et al. 2001). Within the Monte, *E. lobocephalus* is distributed in the Northern Monte and Central Monte (as defined by Roig Juñent and Flores 2001; Rundel et al. 2007). Northern Monte and Central Monte have an annual mean temperature of 13–15° C and annual precipitation of 80–400 mm. Physiognomically, the Monte is a mosaic of two types of vegetation: Shrubby steppes (dominated by species of Zygophyllaceae) and open woodlands of *Prosopis*. The habitat where *E. lobocephalus* was collected (Figure 16) is a thorn dessert dominated by *Larrea divaricata* Cav., *Larrea cuneifolia* Cav. (Zygophyllaceae), *Cassia aphylla* (Cav.) (Leguminosae), and *Prosopis* spp., (Leguminosae). The altitudinal range for the known localities of *E. locephalus* is 450–2500 m.

**Potential distribution of *E. lobocephalus*.** The potential distribution area of *E. lobocephalus* predicted by the model coincides with the area defined as Central Monte and Northern Monte and the Monte-Chaco transition zone as defined by Rundel et al. (2007) and delimitated by Morello (1958) through chorological and ecological criteria. The area in the predicted distribution includes six Argentinean provinces (political): Catamarca, La Rioja, Santiago del Estero, Córdoba (western), San Juan, Mendoza, and a small disjunct area in the Salta province (Figure 15). The potential distribution represents all previous provincial records for *E. lobocephalus* (Martínez 1959) plus Salta and Santiago del Estero where the species has yet to be collected. Label data does not indicate precise localities for Mendoza and no records from this province were used in the model, nevertheless, the species occurrence is predicted for northern Mendoza. Aside from the type series, no records of *E. lobocephalus* were found for Mendoza province.

The Monte is an area with high endemicity (35% for species and 11% for genera based on several orders and families of Insecta) (Roig-Juñent et al. 2001). The dung beetle community in the Monte includes 17 genera and 40 species, with five genera (29%) and 16 species (40%) endemic to the region. Eighteen species (48%) of dung beetles present in the Monte also occur in the Chaco biogeographic province which indicates the close relation these areas have in terms of their dung beetle faunas and presumably reflecting both historical and ecological affinities. Monte, particularly Northern Monte, shares many insect taxa and floristic elements (Roig-Juñent et al.2001) with the Chaco.

**Conservation status.** Protected areas of the Monte have been created to protect landscapes (“Reserva Nacional Valle de la Luna” and Parque Nacional Talampaya” in San Juan and La Rioja respectively), tree populations (“Réserva de la Biósfera Ñacuñán” and “Reserva provincial Telteca” in Mendoza) or some particular species or habitat (“Parque Nacional Lihue Calel” in La Pampa). All 16 reserves in the Monte (1.52% of the total area) are located in the Central or Southern Monte (Roig-Juñent and Claver 1999). These areas are not extensive enough to ensure biodiversity protection and are not close enough to allow biological interchange (Roig-Juñent and Claver 1999). *Ennearabdus lobocephalus* is a species that occurs in low numbers and none of the known distribution localities are included in a protected area. According to Roig-Juñent et al. (2001), based on several insect taxa, the order of importance for conservation priorities for natural areas in the Monte and Chaco is: Northern Monte, Chaco, Central Monte, Southern Monte, Peníinsula Valdez and Uspallata-Caliingasta Valley. This order of importance is consistent with the conservation priorities for *E. lobocephalus* based on the known localities and predicted distribution (Figure 15).
